# Genetic Determinants of Tetracycline Resistance in Clinical *Streptococcus pneumoniae* Serotype 1 Isolates from Niger

**DOI:** 10.3390/antibiotics7010019

**Published:** 2018-03-06

**Authors:** Sani Ousmane, Bouli A. Diallo, Rasmata Ouedraogo

**Affiliations:** 1 Unité de Bactériologie-Virologie, Centre de Recherche Médicale et Sanitaire (CERMES), BP 10887 Niamey, Niger; 2 Faculté des Sciences et Technique, Université Abdou Moumouni, BP 10662 Niamey, Niger; bouli_diallo48@yahoo.fr; 3 Centre Hospitalier Universitaire Pédiatrique Charles de-Gaulle, Ouagadougou, Unité de Formation et de Recherche en Sciences de la Santé, BP 1198 Ouagadougou, Burkina Faso; ramaoued2013@gmail.com

**Keywords:** *Streptococcus pneumoniae*, tetracycline, resistance, genetic determinants

## Abstract

*Streptococcus pneumoniae* serotype 1 is the first cause of pneumococcal meningitis Niger. To determine the underlying mechanism of resistance to tetracycline in serotype 1 *Streptococcus pneumoniae*, a collection of 37 isolates recovered from meningitis patients over the period of 2002 to 2009 in Niger were analyzed for drug susceptibility, and whole genome sequencing (WGS) was performed for molecular analyses. MIC level was determined for 31/37 (83.8%) isolates and allowed detection of full resistance (MIC = 8 µg) in 24/31 (77.4%) isolates. No resistance was found to macrolides and quinolones. Sequence-types deduced from WGS were ST217 (54.1%), ST303 (35.1%), ST2206 (5.4%), ST2839 (2.7%) and one undetermined ST (2.7%). All tetracycline resistant isolates carried a Tn*5253* like element, which was found to be an association of two smaller transposons of Tn*916* and Tn*5252* families. No *tet*(O) and *tet*(Q) genes were detected. However, a *tet*(M) like sequence was identified in all Tn*5253* positive strains and was found associated to Tn*916* composite. Only one isolate was phenotypically resistant to chloramphenicol, wherein a chloramphenicol acetyl transferase (*cat*) gene sequence homologous to *cat*_pC194_ from the *Staphylococcus aureus* plasmid p*C194* was detected. In conclusion, clinical *Streptococcus pneumoniae* type 1 isolated during 2002 to 2009 meningitis surveillance in Niger were fully susceptible to macrolides and quinolones but highly resistant to tetracycline (77.4%) through acquisition of a defective Tn*5253* like element composed of Tn*5252* and Tn*916* transposons. Of the 31 tested isolates, only one was exceptionally resistant to chloramphenicol and carried a Tn*5253* transposon that contained cat gene sequence.

## 1. Introduction

*Streptococcus pneumoniae* (SPN) is the most deadly cause of bacterial meningitis in elderly and <five year old individuals [1,2,3]. In developing countries, the annual incidence of pneumococcal meningitis per 100,000 children varies from 18.1 in Chile to 240 in Gambia [4,5]. In sub-Saharan countries, including Niger, the case fatality rate of pneumococcal meningitis is as high as 50% [6,7]. Pneumococcal meningitis is usually endemic with occasional outbreaks limited to closed institutions like hospitals, long-term care facilities, military camps, childcare centers, or jails [8,9]. Nevertheless, large community outbreaks of meningitis due to SPN serotype 1 were reported, with most characteristics of meningococcal epidemics [10,11]. Vaccination remains the best strategy of control, whilst antibiotics are administered as part of the clinical management of life-threatening infections caused by SPN. However, SPN is a highly recombinogenic bacterium that can easily acquire and incorporate exogenous DNA to adapt to selective pressures including vaccines and antimicrobials [12]. Historically, since the first report of penicillin resistant SPN in Australia in 1967 and later in New Guinea (1974), South Africa (1977), and Spain (1979) pneumococcal resistance, often to multiple antibiotics including tetracycline continues to spread worldwide [13,14]. Discovered in the 1940s, the tetracyclines constitute a family of antibiotics that are broad-spectrum agents, widely used in the therapy of human and animal infections, and at sub therapeutic levels as growth promoters in animal feeds [13]. Three distinct mechanisms of resistance to tetracycline in bacteria have been described and include active efflux, ribosomal protection, and enzymatic drug inactivation. In SPN, tetr is the result of acquisition of one of two genes, the *tet*(M) and less frequently the *tet*(O) genes, both of which encode ribosomal protection proteins [15]. The main source of the *tet*(M) gene is Tn*916,* a conjugative transposon that commonly inserts into the Tn*5252* mobile element to form a composite Tn*5253*-like element [16] which can also carry the chloramphenicol acetyl transferase (cat) genes encoding chloramphenicol resistance [16,17,18,19]. In SPN, acetylation of chloramphenicol is mostly mediated by *cat* gene which was shown to contain sequences homologous to *cat*_pC194_ and other flanking sequences from the *Staphylococcus aureus* plasmid pC194 [20,21].

In a previous study [22], we reported a high level of tetracycline resistance (tetr) and detected *tet*(M) genes among Niger serotype 1 pneumococcal isolates. But, to date, there is very limited molecular data regarding the underlying mechanism of tetr amongst SPN isolates from Niger. In the present study we performed a molecular genome analysis using bio-informatics softwares to comprehend the tetracycline resistance mechanism in SPN1 isolated from 2002 to 2009.

## 2. Results

### 2.1. Patients and Isolates

Isolates were recovered from 37 patients of whom 17 were females (45.9%). Their mean age was 21.8 years with a range of 6 months to 70 years. Thirty-two patients (86.5%) were from Niamey, the capital of Niger, and 5 from other health districts distributed over the country. The period of collection covered 8 years (2002 to 2009) of meningitis surveillance.

### 2.2. Antibiotics Susceptibility Testing

**Figure 1:**
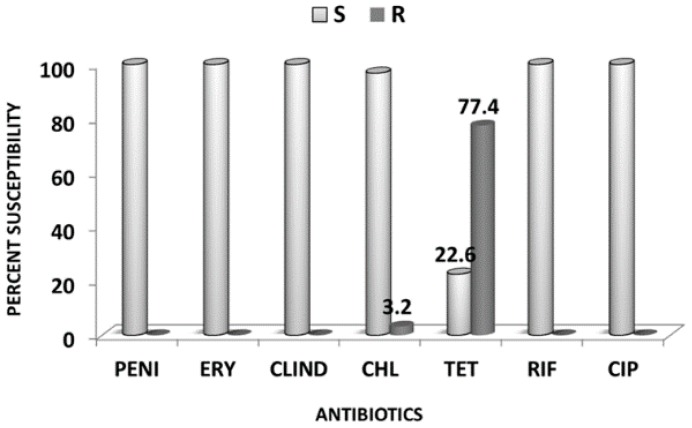
Percent antibiotics susceptibility levels of 31 clinical serotype1 *Streptococcus pneumoniae* recovered from meningitis patients during 2002–2009 meningitis surveillance in Niger. Legend: S = Susceptible; R = Resistant; PENI = Penicillin; ERY= Erythromycin; CLIND = Clindamycin; CHL = Chloramphenicol; TET = Tetracycline; CIP = Ciprofloxacin.

**Figure 2:**
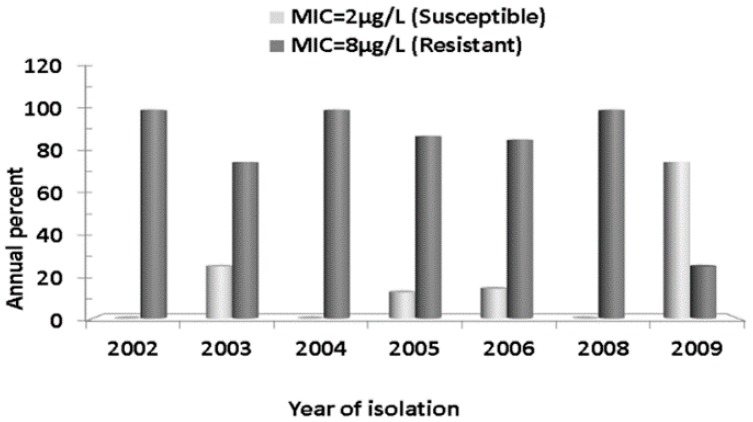
Annual percent distribution of tetracycline resistant *Streptococcus pneumoniae* serotype 1 recovered from meningitis cases from 2002 to 2009 in Niger. The figure shows that resistant isolates prevailed over susceptible ones every year except in 2009. No isolate from 2007 was tested.

Of the 37 isolates sent to NICD, South Africa, 31 (83.8%) remained viable and were tested for drug susceptibility by E-test to determine their Minimum Inhibitory Concentration (MIC) to a panel of antibiotics. All isolates (31/31) were fully susceptible to penicillin, erythromycin, clindamycin, and ciprofloxacin. However, 24/31 (77.4%) isolates were resistant (MIC ≥ 8 µg/L) to tetracycline. Among the tetracycline resistant isolates, one was also additionally resistant to chloramphenicol (Figure 1). Temporal analysis showed that tetracycline resistant isolates remained predominant over those susceptible throughout the study period (Figure 2).

### 2.3. Multi Locus Sequence Typing

Multi Locus Sequence Typing (MLST) allowed detection of 5 sequence types which include ST217 (20/37, 54.1%), ST303 (13/37, 35.1%), ST2206 (2/37, 5.4%), ST2839 (1/37, 2.7%), and undefined (UD) (1/37, 2.7%). Annual distribution indicated that ST217 was the leading cause of serotype 1 pneumococcal meningitis throughout the study period in Niger.

### 2.4. In Silico Detection of Tetracycline Resistance Determinants

#### Tetracycline and Chloramphenicol Resistance Genes

**Figure 3:**
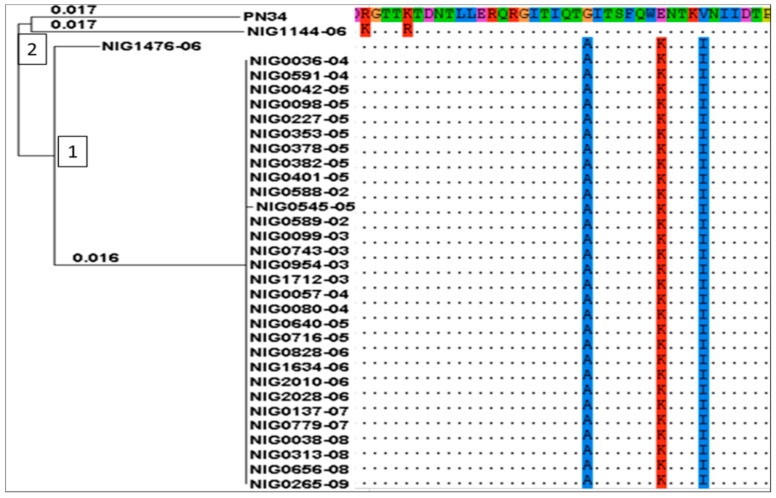
Evolutionary diversity of *tet*(M) genes from clinical serotype 1 *Streptococcus pneumoniae* recovered from meningitis cases between 2002 and 2009 in Niger. The analysis involved 32 *tet*(M) sequences and a reference sequence. PN34 (Accession number: AY466395.1). Sequence names were coded as NIG for Niger followed by isolate identity number, underscore and the year of isolation. Taxa fall in two clades each split into 2 subclades.

Blastn search was conducted for detection of *tet*(O), *tet*(Q) and *tet*(M) genes that code for resistance to tetracycline. No hits were obtained for *tet*(O) and *tet*(Q). However, 32/37 (86.5%) genomes sequences were positive for *tet*(M) including all genomes of those isolates phenotypically resistant to tetracycline. All 32 *tet*(M) sequences were extracted and aligned with a reference gene and a phylogeny tree was constructed using default settings to look into evolutionary divergence. Two major clades of *tet*(M) were observed, each split into two sub-clades (Figure 3). Clade 1 was composed of *tet*(M) genes from all chloramphenicol susceptible but tetracycline resistant strains while, clade 2 was composed of tet(M) from the chloramphenicol resistant strain NIG1144_06 and which aligned with the reference *tet*(M) PN34. Nucleotide sequence analysis revealed that *tet*(M) genes diverge as a result of nucleotide substitutions at different sites compared to sequence the reference *tet*(M) PN34 (accession number AY466395.1). However, these substitutions did not lead to increased or decreased level of resistance between strains. Although only one isolate showed resistance to chloramphenicol by MIC test, we searched across all 37 genomes for the presence of the cat gene coding for chloramphenicol acetyltransferase. Only one hit was obtained from the genome of the strain phenotypically resistant to chloramphenicol. The sequence was 96% identical to *cat*_pC194_ (accession number AAA92251.1) of *Staphylococcus aureus* plasmid pC194.

### 2.5. Carrier Transposons

We searched for carrier transposon with query reference sequence of Tn*5253* (accession number gi|284803504| in the 37 genome database. A total of 32/37 hits were also obtained, one in each *tet*(M) positive genome, indicating that Tn*5253* transposon was the source of *tet*(M) gene. We additionally found that genomes of isolates carrying clade 1 *tet*(M), carried a Tn*5253* element that was located at position 995236..1020197, totaling ~25 kbp spanning towards the genome center. Instead, in genome clade 2 *tet*(M) carrier isolate, Tn*5253* sequence was inserted downstream of the genome on the reverse frame lines and in position 2008480..2062586 (~54.1 kbp). Other analysis showed that in either group, Tn*5253* transposon carried integrases of Tn*916* and Tn*5252* transposons families.

**Figure 4:**
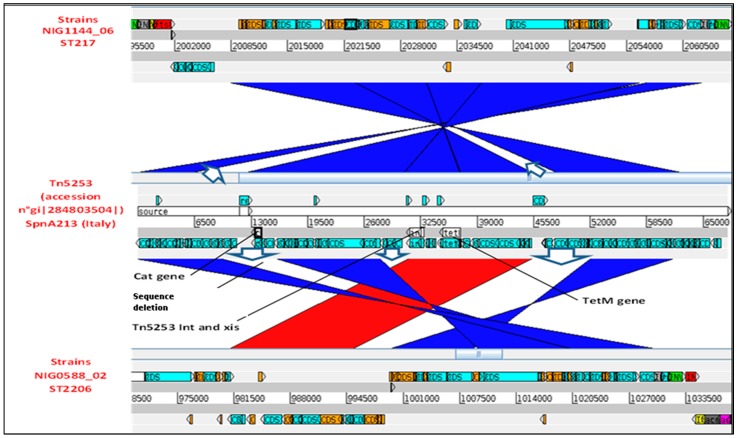
Comparative analysis of Tn*5253* mobile element (accession N° gi|284803504| to Tn*5253* like sequence from genomes of type 1 clinical *Streptococcus pneumoniae* strains NIG0588_02 representing isolates susceptible to chloramphenicol and strain NIG1144_06 that was chloramphenicol resistant.

We compared genomic DNA sequences of isolate NIG0588_02, representing all cat gene negative strains (clade 1) and that of cat gene positive isolate NIG1144_06 (clade 2) to reference sequence of transposon Tn*5253* (accessions number FM201786.3) (Figure 4). We interestingly observed that in both genomes some Tn*5253* sequences were deleted but, specifically, cat gene sequence was absent from the genome of chloramphenicol susceptible strains (clade 1 *tet*(M) carriers). This probably indicates that all chloramphenicol susceptible isolates recovered from 2002 to 2009 were in fact carriers of a defective Tn*5253* transposon that lack cat gene sequence. However, we could find that some coding sequences, including those for cytosine methyltransferase and zeta toxin, were, inserted far away from the main insertion site (not visible on Figure 4).

## 3. Discussion

Tetracyclines are low-cost antibiotics, and consequently are attractive for use in resource poor countries where the majority of people have a limited health care budget, leading to a high level of consumption. Tetracycline is largely used to treat many infections including eye, diarrheal, and urinary infections. Its use in animal feeding is not common in the country but it is readily available in local markets for auto medication and large prescription by medical professionals. This extensive use likely places a strong selective pressure for the development of resistance in pneumococci, which is increasingly reported [23,24].

Serotype 1 SPN is the most important cause of pneumococcal meningitis (43.5%) in Niger [25]. Our study collection consisted of 37 SPN serotype 1 isolates recovered from meningitis cases from 2002 to 2009. The isolates were predominantly (86.5%) recovered from patients residing in Niamey, the capital where the reference laboratory is located. Most CSF specimens collected at peripheral regions of Niamey are sent to the reference laboratory at sub-optimal temperature which makes isolate recovery by culture very difficult or impossible. This explains why the majority of isolates were from Niamey and only a few from other regions of the country.

Whole genome sequencing of our isolate collection allowed detection of four known and one undefined (UD) sequence types. They include ST217 (20/37, 54.1%), ST303 (13/37, 35.1%), ST2206 (2/37, 5.4%), ST2839 (1/37, 2.7%), and UD (1/37, 2.7%). Of these sequence types, ST217 belonging to ST217 clonal complex is one of the common pneumococcal cause of meningitis in sub-Saharan Africa with potential to cause outbreaks [11,26]. Whilst ST217 was an important cause of meningitis in Niger, in Gambia, ST618 was in majority responsible for outbreaks of pneumonia and septicemia from 1997 to 2002 and had led to meningitis outbreak in Burkina Faso between 2002 and 2005 [27,28]. This reflects a geographical clustering of serotype 1 SPN with locally circulating clones rarely shared between countries as previously reported [15].

The present study found a high rate of tetr (77.4%) in serotype 1 SPN based on antimicrobial susceptibility testing. Similar observation was also reported (70.5%) among other pneumococcal serotypes carried by healthy toddlers in Niger in our carriage study [29]. However; a full susceptibility to quinolones and macrolide was observed in the present study and only one isolate was found resistant to chloramphenicol. These results can be a fortunate finding for the management of meningitis cases in the hospitals. Sequence analysis showed that all tetracycline resistant isolates carry *tet*(M) gene to a level as high as 86.5% (32/37 isolates). Additionally, sequence of Tn*5253*-like genetic element were detected which were reported to also select for resistance to other antimicrobials like chloramphenicol [16,17,18,19]. Interestingly, a comparative analysis showed that all chloramphenicol susceptible isolates were carriers of but a defective Tn5*253* like transposon that lacked cat gene sequence, a finding that agrees with a previous report [28]. Nevertheless, this resistance mechanism needs to be continuously monitored to follow antimicrobial resistance of pneumococci in Niger.

Observation of coding sequences carried by the Tn*5253*-like element in both chloramphenicol resistant and susceptible isolates revealed the presence of open reading frames for both Tn*5252* and Tn*916* transposons including those coding for integrase and excisionase. This indicated that the mega Tn*5253*-like element was in fact a composite of these two smaller transposons as was described somewhere [17].

## 4. Materials and Methods

We included all clinical serotype 1 SPN isolates available in the collection of *Centre de Recherche Medical et Sanitaire*, CERMES, a national reference laboratory for meningitis surveillance in Niger. The collection comprised of 37 isolates recovered from CSF during national meningitis surveillance from 2002 to 2009. Culture was made using fresh Columbia blood agar medium supplemented with 5–7% sheep blood followed by incubation at 35–37 °C for 18 to 24 h under CO_2_ atmosphere (GenBoxCO2, BioMerieux, St. Louis, MO, USA). The isolates were subjected to Illumina sequencing at the Welcome Trust Sanger Institute, genome assembly and *in silico* MLST profiling by the Pneumococcal African Genomics Consortium (http://www.pagegenomes.org) as described by Chaguza et al. 2015 [12]. In total, 31/37 (83.8%) isolates have been successfully re-awoken and their MIC’s determined by broth microdilution using commercially prepared Trek panels (Trek Diagnostics Inc., Westlake, OH, USA) containing Mueller–Hinton broth supplemented with 5% lysed horse blood at the National Institute for Communicable Diseases (NICD), South Africa. MIC interpretation was based on CLSI guidelines 2011 [30]. Sequence analysis was carried out at CERMES by use of bioinformatics tools including standalone NCBI-BLAST-2.2.29+ for sequences searching; CLC Genomics Workbench 9.0 (limited version) by QIAGEN for sequence and annotation viewing and MEGA.7.0.26 for alignment and phylogenic tree construction using default settings. A phylogenetic tree of the *tet*(M) genes was inferred by computing evolutionary distances using the Nei–Gojobori method. A local database consisting of 37 concatenated genomic DNA sequences was built for sequences searching. Nucleotide BLAST hits with high score, an e-value of 0.0 and an identity percentage of at least 96% were considered significant. Sequences comparison was carried out with Artemis and Artemis Comparison Tool (ACT) release 12.0.0 (2013). Reference sequences including *tet*(M) PN34 (accession number AY466395.1), *tet*(O) (accession number Y07780.1), Tn*916* (accession number U09422.1), Tn*5253* (accessions number FM201786.3), *catQ* (accession number M55620.1) and *cat*_pC194_ (accession number AAA92251.1) were used as query sequences.

### Ethics Statement

Pneumococcal isolates were obtained from the collection of the *Centre de Recherche Médicale Sanitaire,* CERMES, a national reference laboratory (NRL) for the surveillance of meningitis in Niger. For this study, no additional sample or data collection was made. Shipment of the isolates and the extracted DNA to partners for processing and analysis was approved by the Ministry of Health.

## 5. Conclusions

A high level of tetracycline resistance (>77%) essentially mediated by *tet*(M) gene in clinical serotype 1 *Streptococcus pneumoniae* from Niger was observed as previously reported. All resistant isolates were found to carry a Tn*5253*-like element that fortunately did not carry multiple antibiotic resistant determinants. However, these data indicate that a continuous surveillance of antimicrobial resistance is necessary in Niger.
